# Supplementary figures and images for: Dilution and titration of cell-cycle regulators may control cell size in budding yeast
Source: PLoS Comput Biol. 2018 Oct 24;14(10):e1006548. doi: 10.1371/journal.pcbi.1006548 (PMC6218100; doi:10.1371/journal.pcbi.1006548)

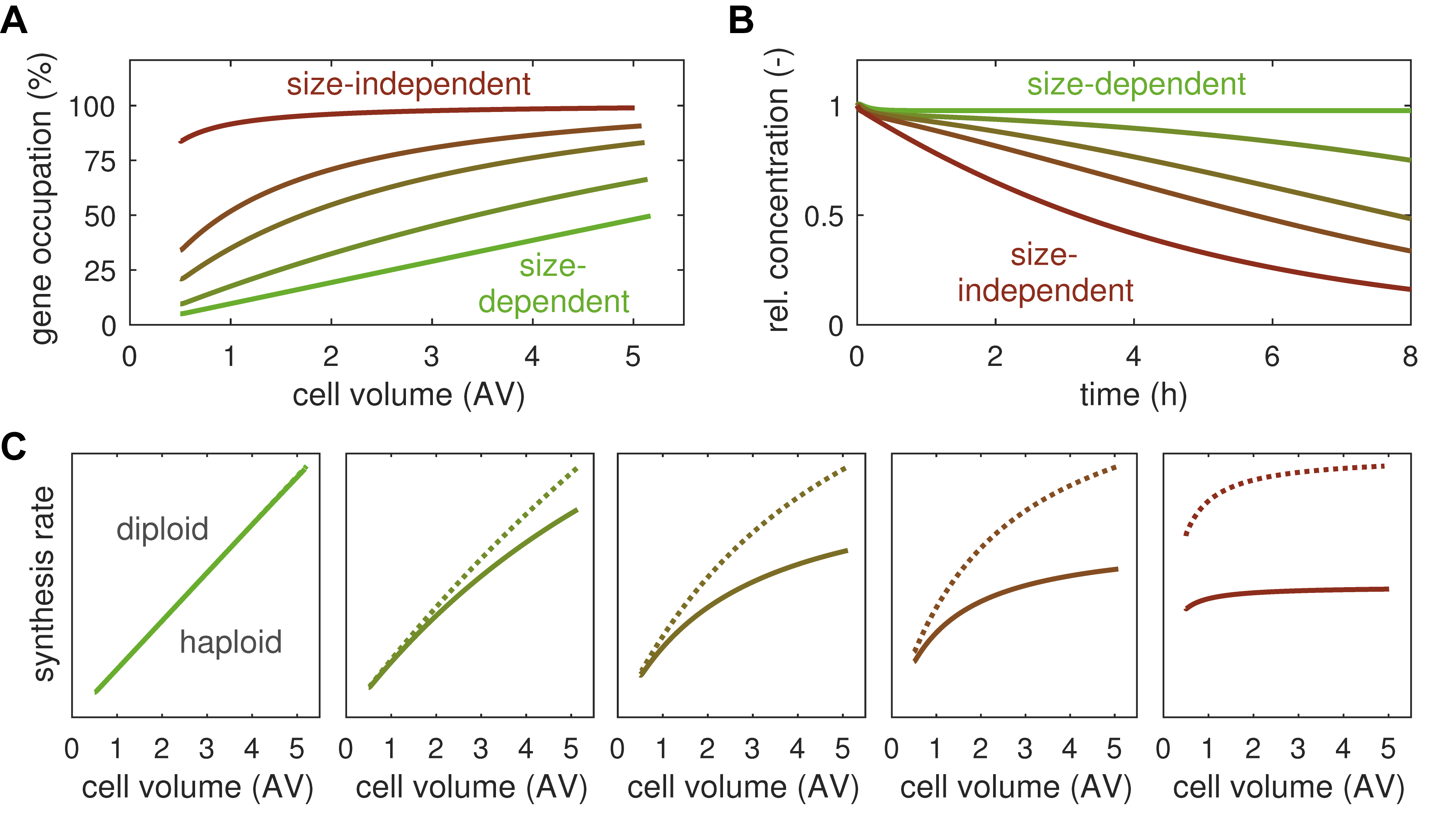

Supplement: S1 Fig — Expression patterns of genes with different equilibrium constants for TM binding, ranging from high (size-independent) to low (size-dependent) affinity. (A) Gene occupation by TM in dependence on cell volume. (B) Relative protein concentration (normalised to initial concentration) in dependence on time in a growing cell. (C) Protein synthesis rates in haploid (solid) and diploid (dashed) cells. Curves in left panel overlap. (TIF) [file pcbi.1006548.s004.tif]

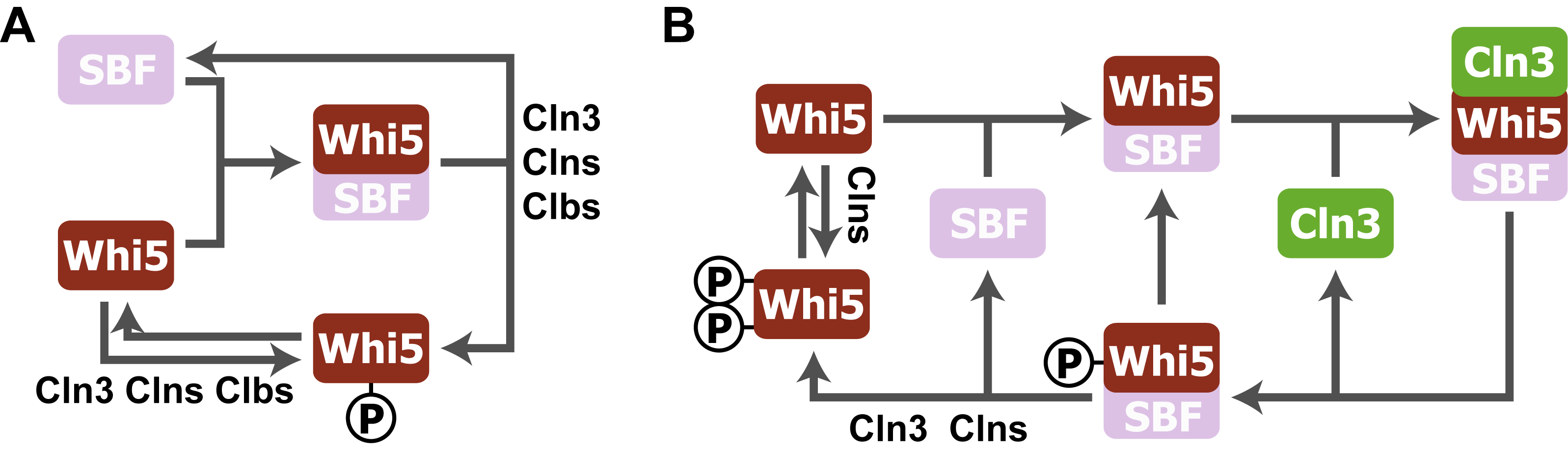

Supplement: S2 Fig — (A) Scheme of SBF inhibition in the inhibitor-dilution model. Whi5 strongly binds to SBF in a concentration-based manner, causing SBF inhibition. Both free and complexed Whi5 can be phosphorylated by Cln3, Cln1/2 and Clb1/2. Phosphorylation of SBF:Whi5 complexes leads to their dissociation, which activates SBF. (B) Scheme of SBF inhibition in the titration model. Whi5 strongly binds to SBF, which occupies a fixed number of nuclear sites. Cln3 strongly binds to Whi5:SBF, slowly hypo-phosphorylating the complex and dissociating in the process. Hypo-phosphorylated Whi5:SBF can return to the unphosphorylated state. However, when free Cln3 or Cln1/2 are available, Whi5 becomes hyper-phosphorylated leading to Whi5 dissociation and SBF activation. Subsequently, the free pool of Whi5 is phosphorylated by Cln1/2. Note that in both models, active SBF drives the synthesis of Cln1/2, which accelerates Whi5 phosphorylation and SBF activation (see Fig 2A). This positive feedback establishes an abrupt toggle switch at Start. (TIF) [file pcbi.1006548.s005.tif]

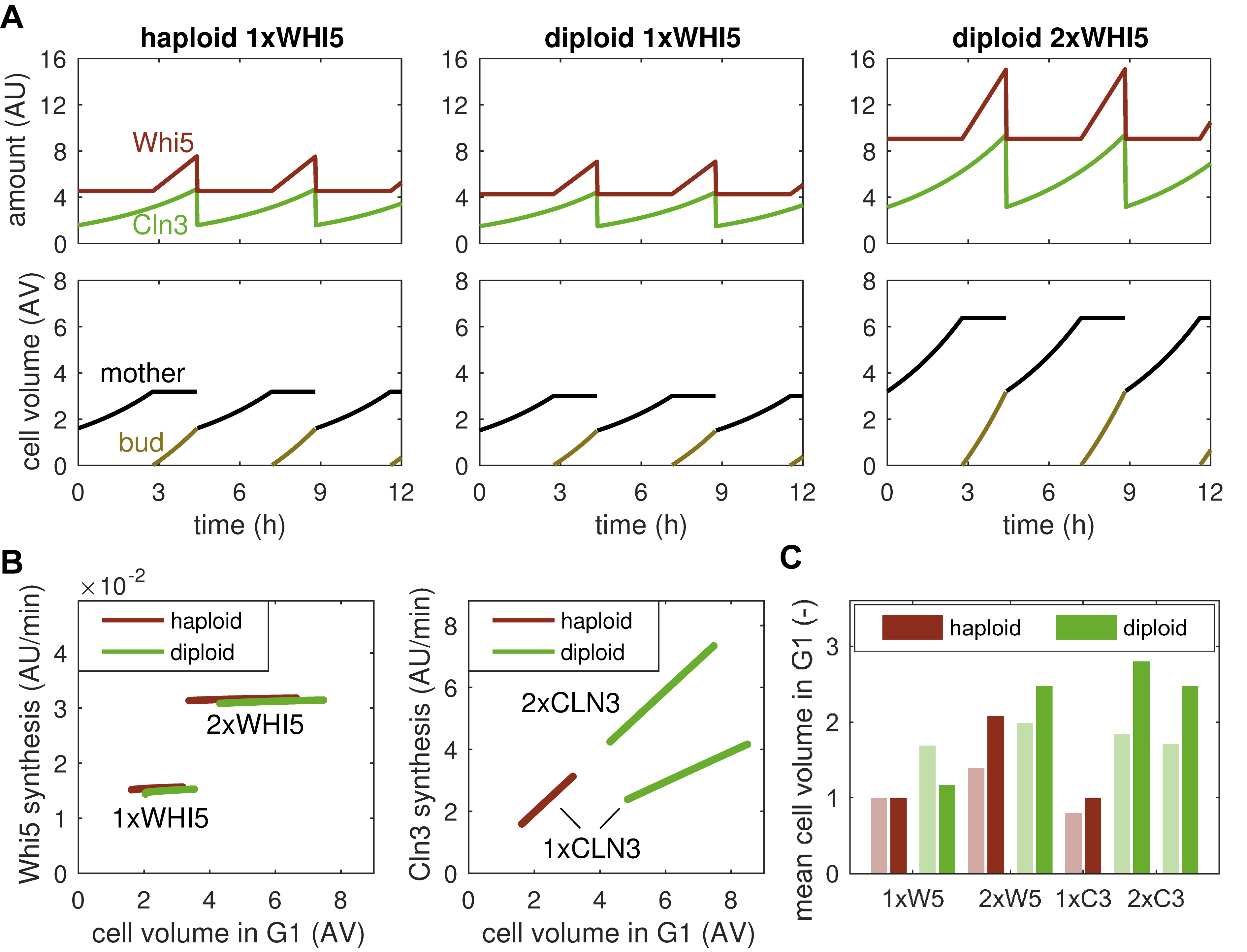

Supplement: S3 Fig — (A) Amount of Whi5 and Cln3 (upper panels) and cell volume (lower panels) in haploid cells with one WHI5 copy (left), diploid cells with one WHI5 copy (middle) and diploid cells with two WHI5 copies (right). Note the increase in Whi5 synthesis (increased slope during synthesis period) and cell volume in the latter case. (B, C) Same as in Fig 3B and 3C except that the S/G2/M duration of all diploid cells was increased by approximately 10% based on experiments in Ref. [13]. (TIF) [file pcbi.1006548.s006.tif]

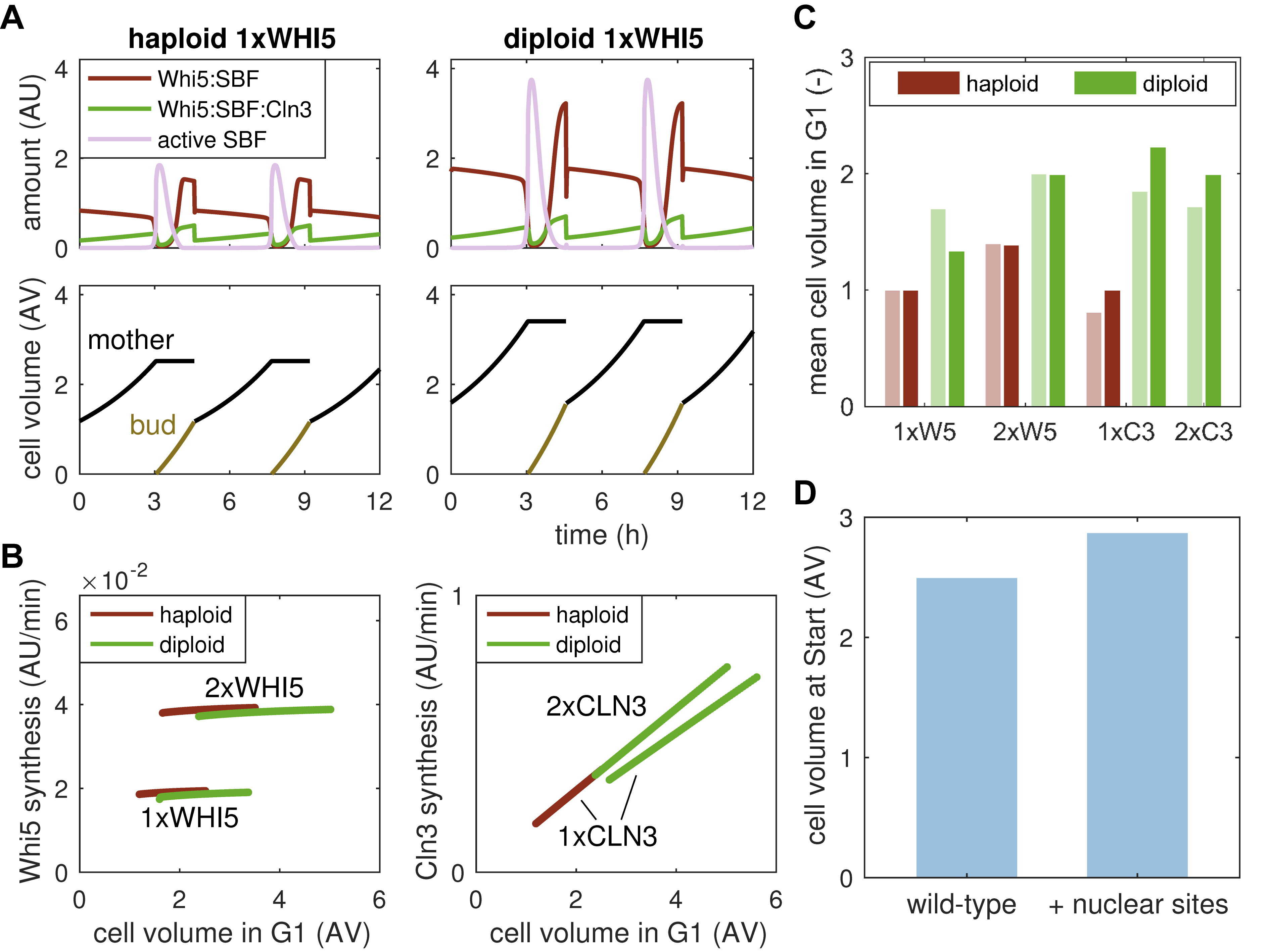

Supplement: S4 Fig — (A) Amount of Whi5:SBF, Whi5:SBF:Cln3 and active SBF (upper panels), and cell volume (lower panels) in haploid (left) and diploid (right) cells with one WHI5 copy in the titration model. Note the increase in cell volume for diploid cells due to the presence of twice the number of SBF complexes on binding sites (sum of the three species shown). (B, C) Same as in Fig 4E and 4F except that Cln3 synthesis in diploid cells with one CLN3 was manually increased by a factor of 0.7. (D) Simulated cell size at Start for a normal haploid cell (wild-type) and a haploid cell harbouring a plasmid that contains SBF binding sites (+ nuclear sites) following the experiment in Fig 7 of Ref. [20]. The total number of binding sites was increased by ~30%. (TIF) [file pcbi.1006548.s007.tif]

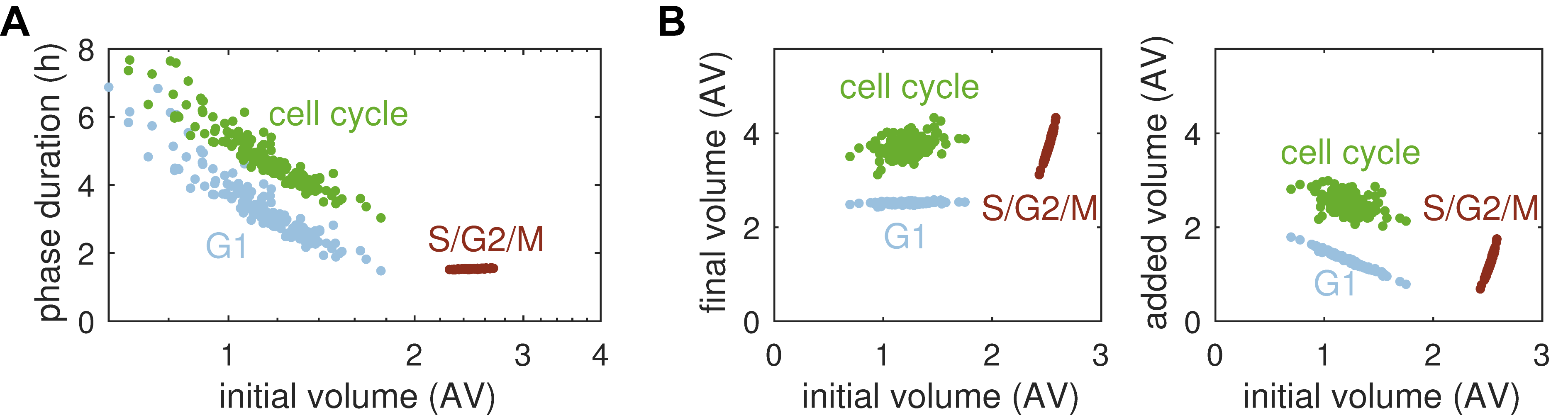

Supplement: S5 Fig — (A) Duration of the indicated cell cycle phase or the whole cycle with respect to volume at the beginning of the phase for the simulations in Fig 5. Note the logarithmic scaling of the x-axis. (B) Same as in Fig 5B, except that the amount of Whi5 at cell birth was manually set to a constant, birth-size-independent value. This results in an almost ideal G1 sizer (slope of -0.95 for volume added in G1 versus birth size). Note that the phenomenological adder over the whole cell cycle disappears in this case (slope of -0.49 for volume added over the whole cell cycle versus birth size). (TIF) [file pcbi.1006548.s008.tif]

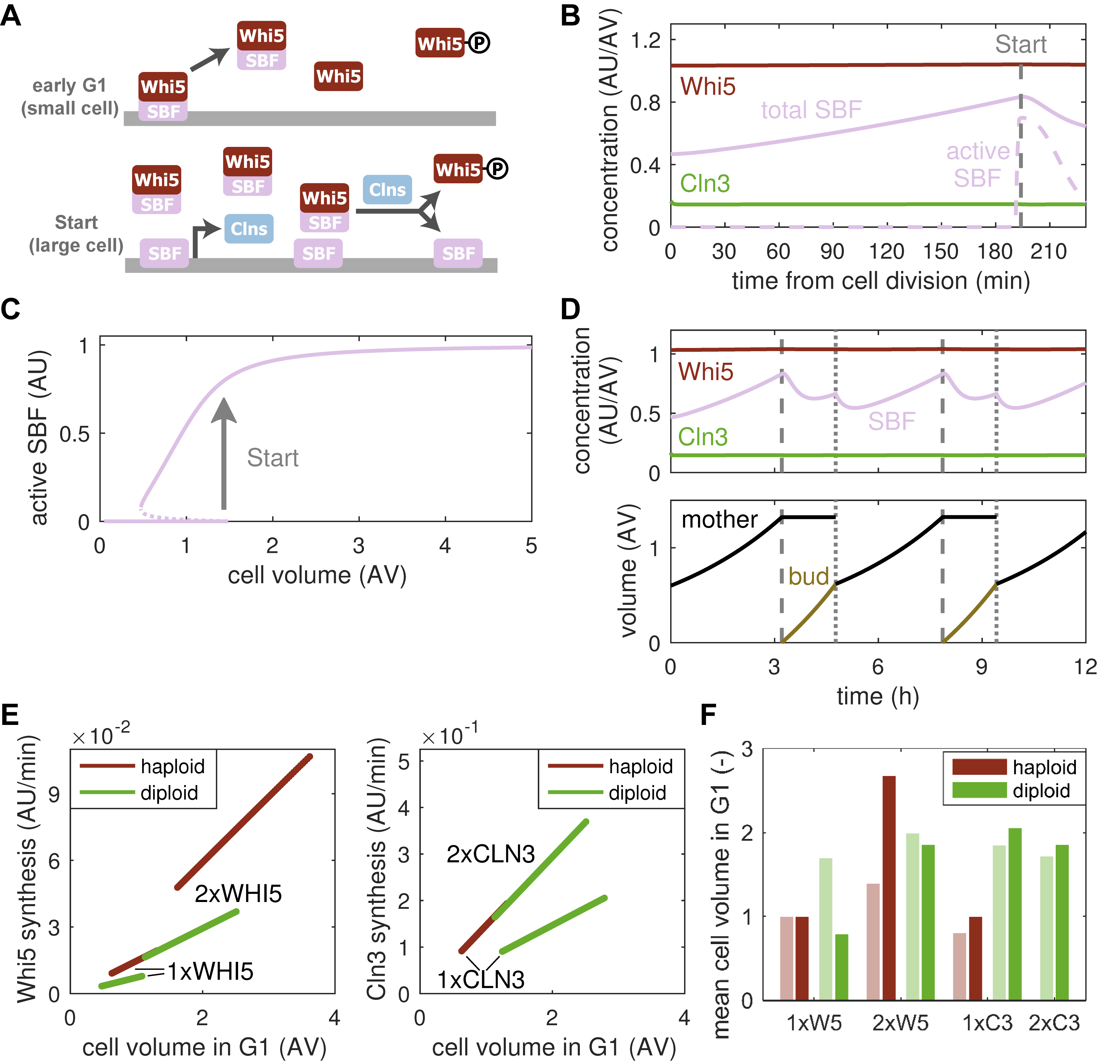

Supplement: S6 Fig — (A) Schematic of the SBF-increase model. In early G1, Whi5 outnumbers SBF and prevents its activation. A fraction of Whi5 is phosphorylated by Cln3 and does not participate in inhibition. As cells grow, the SBF concentration increases such that SBF is able to overcome inhibition and induce Cln1 and Cln2 synthesis. Whi5 phosphorylation then liberates the rest of the SBF pool. (B) Concentration of Whi5 and Cln3 as well as total and active SBF in a growing cell. Vertical dashed line marks Start. (C) Stable (solid) and unstable (dashed) steady states of active SBF with respect to cell volume in the SBF-increase model. Arrow indicates Start transition. (D) Concentration of cell cycle regulators (top) and cell volume (bottom) over multiple generations. Dashed and dotted lines mark Start and division, respectively. (E) Simulation of Whi5 and Cln3 synthesis rates in haploid and diploid cells with the indicated copy number of WHI5 and CLN3. (F) Mean cell volume in G1 for data in [13] (light bars) and simulations in E (dark bars). Values were normalized to haploid cells with one WHI5 copy for each case. (TIF) [file pcbi.1006548.s009.tif]
