# Supplementary material for: Dilution and titration of cell-cycle regulators may control cell size in budding yeast
Source: PLoS Comput Biol. 2018 Oct 24;14(10):e1006548. doi: 10.1371/journal.pcbi.1006548 (PMC6218100; doi:10.1371/journal.pcbi.1006548)
Supplement: S1 Text — (DOCX) [file pcbi.1006548.s010.docx]

## S1 Text. Differences between inhibitor-dilution and titration model

The main differences between the models for inhibitor dilution and titration of nuclear sites pertain to the interactions of Whi5, SBF and Cln3. Below we describe in detail how cell volume growth promotes the Start transition in each of these models and where the size-dependent biochemical signal that couples cell cycle progression to growth originates from. Note that the equations below are simplified versions of the equations used in the models and are for illustrative purposes only.

In the inhibitor-dilution model, both Cln3 and SBF are size-dependent proteins that increase in number as the volume grows (they remain constant in concentration), while Whi5 is a size-independent protein with constant molecule number (decreasing concentration). All three proteins react in a concentration-dependent manner. In order for cell size to affect the Start transition, SBF activity needs to change with cell volume, which can result, in principle, either from a size-dependent phosphorylation of Whi5 by Cln3 or from a size-dependent complex formation of Whi5 and SBF. With respect to the first case, a general equation for the phosphorylation of Whi5 ($WHI$) by Cln3 ($CLN3$) in terms of their molecule numbers is given by

$$\frac{d\left( WHI_{p} \right)}{dt}=\frac{k^{\mathrm{Ph}}}{V}\cdot CLN3\cdot WHI-k^{\mathrm{Dp}}\cdot WHI_{p},$$

where $k^{\mathrm{Ph}}$ and $k^{\mathrm{Dp}}$ denote the phosphorylation and dephosphorylation rate, respectively, and $WHI_{p}$ is the number of phosphorylated Whi5 molecules. The total number of Whi5 molecules ($WHI_{t}$) is

$$WHI_{t}=WHI+WHI_{p},$$

yielding

$$\frac{d\left( WHI_{p} \right)}{dt}=\frac{k^{\mathrm{Ph}}}{V}\cdot CLN3\cdot\left( WHI_{t}-WHI_{p} \right)-k^{\mathrm{Dp}}\cdot WHI_{p}.$$

Therefore, the steady state of $WHI_{p}$ ($\bar{WHI_{p}}$) follows as

| $\bar{WHI_{p}}=\frac{k^{\mathrm{Ph}}\cdot WHI_{t}\cdot CLN3/V}{k^{\mathrm{Dp}}+k^{\mathrm{Ph}}\cdot CLN3/V}.$ | (51) |
| --- | --- |

We note that the concentration of Cln3 ($CLN3/V$) and the total number of Whi5 molecules ($WHI_{t}$) are constant since Cln3 and Whi5 are size-dependent and size-independent proteins, respectively. Hence, neither the number of phosphorylated nor unphosphorylated Whi5 molecules changes as the cell volume increases and the Cln3 concentration merely determines the fraction of phosphorylated Whi5

$$\frac{\bar{WHI_{p}}}{WHI_{t}}=\frac{k^{\mathrm{Ph}}\cdot CLN3/V}{k^{\mathrm{Dp}}+k^{\mathrm{Ph}}\cdot CLN3/V}.$$

In other words, the dilution of Whi5 against Cln3 does not result in a size-dependent phosphorylation state of Whi5 that could promote the Start transition in response to a cell volume increase. Intuitively, the increase in volume makes it harder for Cln3 to find and phosphorylate Whi5, which is exactly compensated for by an increase in the number of Cln3 molecules. Note that this also holds for Cln3-Whi5 interactions in the nucleus since the nuclear volume in budding yeast growth proportionally to the overall cell volume [48].

For the second case, the binding of Whi5 to SBF ($SBF$), we get the following general equation

$$\frac{d\left( WHISBF \right)}{dt}=\frac{k^{\mathrm{As}}}{V}\cdot SBF\cdot WHI-k^{\mathrm{Ds}}\cdot WHISBF,$$

where $k^{\mathrm{As}}$ and $k^{\mathrm{Ds}}$ are the association and dissociation rates, respectively, and $WHISBF$ is the SBF:Whi5 complex. In this case, the conservation equations for total Whi5 ($WHI_{t}$) and total SBF (${SBF}_{t}$) are

$WHI_{t}=WHI+WHISBF,$

$${SBF}_{t}=SBF+WHISBF.$$

We hence get

$$\frac{d\left( WHISBF \right)}{dt}=\frac{k^{\mathrm{As}}}{V}\cdot\left( SBF_{t}-WHISBF \right)\cdot\left( WHI_{t}-WHISBF \right)-k^{\mathrm{Ds}}\cdot WHISBF.$$

To calculate the steady state of $WHISBF$ ($\bar{WHISBF}$) we need to solve the quadratic equation

$$0=\bar{WHISBF}^{2}-\left( SBF_{t}+WHI_{t}+\frac{k^{\mathrm{Ds}}}{k^{\mathrm{As}}}V \right)\cdot\bar{WHISBF}+SBF_{t}\cdot WHI_{t}.$$

Since Whi5 is a stoichiometric inhibitor of SBF, we can assume that it binds strongly to its target and hence

$k^{\mathrm{Ds}}\ll k^{\mathrm{As}}$ giving $\frac{k^{\mathrm{Ds}}}{k^{\mathrm{As}}}V\approx0$.

Using the quadratic formula and the conservation equation for SBF yields the steady state of active SBF ($\bar{SBF}$) as

$$\bar{SBF}=SBF_{t}-\bar{WHISBF}=SBF_{t}-\frac{1}{2}\left( SBF_{t}+WHI_{t}\pm\sqrt{\left( SBF_{t}+WHI_{t} \right)^{2}-4\cdot SBF_{t}\cdot WHI_{t}} \right).$$

In the limiting case of strong SBF:Whi5 binding, we thus obtain

| $\bar{SBF}=\left\{ \begin{matrix} 0 & \mathrm{for} & SBF_{t}<WHI_{t} \\ SBF_{t}-WHI_{t} & \mathrm{for} & SBF_{t}\geq WHI_{t} \end{matrix} \right..$ | (52) |
| --- | --- |

Therefore, SBF will be inhibited as long as there are enough Whi5 molecules to form SBF:Whi5 complexes, and free (active) SBF becomes available when the total SBF number exceeds that of Whi5. Note that since SBF is a size-dependent protein, $SBF_{t}$ increases in number as the cell volume growth, while $WHI_{t}$ remains constant. Hence, in the inhibitor-dilution model, cell volume drives the activation of SBF (the Start transition) by an increase in the number of SBF molecules over Whi5 molecules. Phosphorylation by Cln3 can reduce the total number of Whi5 molecules that are available for complexes formation but does not do so in a size-dependent manner.

In the titration model, the molecule numbers of Whi5 and Cln3 are again assumed to stay constant and increase, respectively, during cell volume growth. However, since there is a limited number of SBF binding sites on the genome and only the SBF that is bound to these sites affects Start, the effective total number of SBF molecules ($SBF_{t}$) now remains constant as the cell volume increases. Using similar arguments as above (in particular strong Whi5-SBF binding), the level of active SBF (SBF not inhibited by Whi5) follows Eq. 52. Since small cells do not immediately progress through Start, one has to assume that the amount of Whi5 present in these cells is sufficiently large to inhibit all SBF on binding sites, i.e., $WHI_{t}>SBF_{t}$, and thus

$\bar{SBF}=0$ and $\bar{WHISBF}=SBF_{t}$.

These levels, including the level of active SBF, do not change with cell volume because both $WHI_{t}$ and $SBF_{t}$ are constant. Hence, Whi5:SBF complex formation does not provide a size-dependent signal in the titration model. For the phosphorylation of Whi5 by Cln3, we invoked the titration argument proposed in Ref. [20], assuming that Cln3 binds to Whi5:SBF complexes in order to hypo-phosphorylate Whi5. The level of Cln3:Whi5:SBF complexes ($CLN3WHISBF$) is thus given by

$$\frac{d\left( CLN3WHISBF \right)}{dt}=\frac{k^{\mathrm{As}}}{V}\cdot CLN3\cdot WHISBF-{(k}^{\mathrm{Ph}}+k^{\mathrm{Ds}})\cdot CLN3WHISBF,$$

where $k^{\mathrm{As}}$ and $k^{\mathrm{Ds}}$ are the association and dissociation rate, respectively, and we assumed that Whi5 phosphorylation with rate $k^{\mathrm{Ph}}$ leads to complex dissociation. In the limiting case of strong Cln3 binding to Whi5:SBF and relatively slow hypo-phosphorylation, the steady-state number of Cln3:Whi5:SBF complexes ($\bar{CLN3WHISBF}$) is thus given by

| $\bar{CLN3WHISBF}=\left\{ \begin{matrix} CLN3_{t} & \mathrm{for} & CLN3_{t}<WHISBF_{t} \\ WHISBF_{t} & \mathrm{for} & CLN3_{t}\geq WHISBF_{t} \end{matrix} \right..$ | (53) |
| --- | --- |

Similarly, the steady-state amount of free Cln3 ($\bar{CLN3}$) is

| $\bar{CLN3}=\left\{ \begin{matrix} 0 & \mathrm{for} & CLN3_{t}<WHISBF_{t} \\ CLN3_{t}-WHISBF_{t} & \mathrm{for} & CLN3_{t}\geq WHISBF_{t} \end{matrix} \right..$ | (54) |
| --- | --- |

In other words, as the number of Cln3 molecules increases with cell size, Cln3 first fills up the Whi5:SBF complexes on SBF binding sites before free Cln3 emerges. Theoretically, a size-dependent signal can be generated by both processes: the hypo-phosphorylation of Whi5, which is proportional to $\bar{CLN3WHISBF}$, and the release of free Cln3, which can drive Start directly by hyper-phosphorylating Whi5 and liberating SBF. The later process would be particularly suitable since free Cln3 is an ultrasensitive function of volume, i.e., it increases suddenly as $CLN3_{t}$ exceeds the number of Whi5:SBF complexes. Such ultrasensitivity (in this case called ‘stoichiometric-inhibitor ultrasensitivity’ [49]) is one prerequisite for a bistable transition like Start.

In summary, the size-dependent signal that controls progression through Start originates from SBF accumulation against a constant number of Whi5 molecules in our inhibitor-dilution model, and from Cln3 accumulation against a constant number of Whi5:SBF complexes on nuclear sites in our titration model.

## References

48. Jorgensen P, Edgington NP, Schneider BL, Rupeš I, Tyers M, Futcher B, et al. The Size of the Nucleus Increases as Yeast Cells Grow. Mol Biol Cell. 2007;18: 3523–3532. doi:10.1091/mbc.e06-10-0973

49. Ferrell JE Jr, Ha SH. Ultrasensitivity part II: multisite phosphorylation, stoichiometric inhibitors, and positive feedback. Trends Biochem Sci. 2014;39: 556–569. doi:10.1016/j.tibs.2014.09.003
