# Supplementary material for: Dilution and titration of cell-cycle regulators may control cell size in budding yeast
Source: PLoS Comput Biol. 2018 Oct 24;14(10):e1006548. doi: 10.1371/journal.pcbi.1006548 (PMC6218100; doi:10.1371/journal.pcbi.1006548)
Supplement: S2 Text — (DOCX) [file pcbi.1006548.s011.docx]

**S2 Text. Model for increasing SBF concentration**

In a recent study, Dorsey et al. determined the absolute concentrations of the G1/S transcription factors SBF and MBF, the transcriptional repressor Whi5, and of Cln1 and Cln2 [35]. Instead of a decrease in Whi5 concentration they describe a size-dependent increase in the concentration of Swi4, a component of SBF, causing a decrease in the Whi5/Swi4 ratio in large cells. In order to model this hypothesis, we used the wiring diagram in Fig. 2A and assumed that Whi5 is a size-dependent protein, which remains constant in concentration. The increase in SBF concentration was modelled by assuming that SBF synthesis is size-dependent, while SBF degradation is facilitated by a size-independent protein. Cell growth dilutes this protein causing an overall increase in SBF concentration. As a result, the inhibitor Whi5 outnumbers SBF in small cells, but SBF is able to overcome inhibition as cells grow (S6A and S6B Figs.). Note that a constant fraction of Whi5 is kept phosphorylated by Cln3 reducing the pool of Whi5 that is available for inhibition. As in the other models, positive feedback via Cln1 and Cln2 establishes a bistable size checkpoint (S6C Fig.) that leads to rapid SBF activation once a critical cell size is reached. This checkpoint facilitates cell size homeostasis over multiple generations (S6D Fig.).

When simulating gene copy-number and ploidy effects, the model correctly reproduces Cln3 synthesis rates and the corresponding changes in cell size (S6E Fig., right panel). In particular, diploid cells are twice the size of haploid cells. Note that this prediction depends on our assumption of SBF degradation being mediated by a size independent protein. Since the degrading protein is size-independent, an additional genome copy causes its expression to double. Hence, the diploid cell needs to reach twice the size to produce the same number of SBF molecules, resulting in a doubling of the critical size at Start. In contrast to experimental results (see Fig. 3A), Whi5 synthesis rates in the model increase with cell size (S6E Fig.) and the model is unable to correctly capture the size of diploid cells with one *WHI5* copy and haploid cells with two *WHI5* copies (S6F Fig.). In the former case, a second genome copy reduces SBF levels by half, which should increase cell size. But this effect is compensated by a reduction of the Whi5 concentration due to a missing *WHI5* copy. Hence, diploid cells with one *WHI5* copy are almost the same size than haploid cells. For haploid cells with two *WHI5*, an increased Whi5 expression leads to cells that are larger than diploid cells contrary to experimental observations.
