# Supplementary material for: Dilution and titration of cell-cycle regulators may control cell size in budding yeast
Source: PLoS Comput Biol. 2018 Oct 24;14(10):e1006548. doi: 10.1371/journal.pcbi.1006548 (PMC6218100; doi:10.1371/journal.pcbi.1006548)
Supplement: S1 Table — (DOCX) [file pcbi.1006548.s012.docx]

| **S1 Table. Parameters used in both size-control models.** | | | |
| --- | --- | --- | --- |
| **Parameter** | **Description** | **Value** | **Unit**^a^ |
| $GCN$ | gene copy number (ploidy) | 1^b^ | molecules |
| $GC_{t}$ | copy number of Cln3 gene | 1^b^ | molecules |
| $GD_{t}$ | total number of size-dependent genes | 500^b^ | molecules |
| $GW_{t}$ | copy number of Whi5 gene ($=GI_{t}$) | 1^b^ | molecules |
| $Gr_{d}$ | binary variable for daughter cell growth | 0 or 1 | − |
| $Gr_{m}$ | binary variable for mother cell growth | 0 or 1 | − |
| $j_{\mathrm{Cdc}}$ | Michaelis-Menten constant for Cdc20 (in-)activation | 10^-3^ | AU/AV |
| $j_{\mathrm{Cdh}}$ | Michaelis-Menten constant for Cdh1 (in-)activation | 10^-3^ | AU/AV |
| $j_{\mathrm{Clb}}^{\mathrm{Sy}}$ | Michaelis-Menten constant for Clb1/2 synthesis | 0.3 | AU/AV |
| $k_{\mathrm{CdcClb}}^{\mathrm{Ac}}$ | Clb1/2-dependent Cdc20 activation | 0.5 | 1/min |
| $k_{\mathrm{Cdh}}^{\mathrm{Ac}}$ | constitutive Cdh1 activation | 0.01 | AU/(AV$\cdot$min) |
| $k_{\mathrm{CdhCdc}}^{\mathrm{Ac}}$ | Cdc20-dependent Cdh1 activation | 2 | 1/min |
| $k_{\mathrm{GdTm}}^{\mathrm{As}}$ | association of size-dependent genes and TM | 1 | AV/(molecule$\cdot$min) |
| $k_{\mathrm{GiTm}}^{\mathrm{As}}$ | association of size-independent genes and TM | 10 | AV/(molecule$\cdot$min) |
| $k_{\mathrm{Clb}}^{\mathrm{De}}$ | constitutive Clb1/2 degradation | 0.01 | 1/min |
| $k_{\mathrm{ClbCdh}}^{\mathrm{De}}$ | Cdh1-dependent Clb1/2 degradation | 2 | AV/(AU$\cdot$min) |
| $k_{\mathrm{Cln}}^{\mathrm{De}}$ | constitutive Cln1/2 degradation | 1 | 1/min |
| $k_{Cln3}^{\mathrm{De}}$ | Cln3 degradation | 1 | 1/min |
| $k_{\mathrm{Sbf}}^{\mathrm{Dp}}$ | dephosphorylation of SBF | 0.2 | 1/min |
| $k_{\mathrm{Whi}}^{\mathrm{Dp}}$ | dephosphorylation of Whi5 | 1 | 1/min |
| $k_{\mathrm{GdTm}}^{\mathrm{Ds}}$ | dissociation of TM from size-dependent genes | 1 | 1/min |
| $k_{\mathrm{GiTm}}^{\mathrm{Ds}}$ | dissociation of TM from size-independent genes | 0.1 | 1/min |
| $k_{\mathrm{Cdc}}^{\mathrm{In}}$ | constitutive Cdc20 inactivation | 0.25 | AU/(AV$\cdot$min) |
| $k_{\mathrm{CdhClb}}^{\mathrm{In}}$ | Clb1/2-dependent Cdh1 inactivation | 2 | 1/min |
| $k_{\mathrm{CdhCln}}^{\mathrm{In}}$ | Cln1/2-dependent Cdh1 inactivation | 0.125 | 1/min |
| $k_{\mathrm{SbfClb}}^{\mathrm{Ph}}$ | Clb1/2-dependent SBF phosphorylation | 5 | AV/(AU$\cdot$min) |
| $k_{\mathrm{Clb}}^{\mathrm{Sy}}$ | constitutive Clb1/2 synthesis | 0.01 | AU/(molecule$\cdot$min) |
| $k_{\mathrm{ClbClb}}^{\mathrm{Sy}}$ | Clb1/2-dependent Clb1/2 synthesis | 0.3 | AU/(molecule$\cdot$min) |
| $k_{\mathrm{Cln}}^{\mathrm{Sy}}$ | Cln1/2 synthesis | 8 | AU/(molecule$\cdot$min) |
| $k_{\mathrm{TM}}^{\mathrm{Sy}}$ | synthesis of transcription machinery | 2.1 | 1/min |
| $k_{\mathrm{Vo}}^{\mathrm{Sy}}$ | cell volume growth | 0.042 | AV/(molecule$\cdot$min) |
| $StartThr$ | Cln1/2 threshold for Start transition | 0.3 | AU/AV |
| $MitosisThr$ | Cln1/2 + Clb1/2 threshold for cell division | 0.2 | AU/AV |

^a^AU, arbitrary unit of number of molecules; AV, arbitrary unit of cell volume.

^b^Parameters corresponding to a haploid cell. Changes made for ploidy mutants are listed in S5 Table.
