# Supplementary material for: Dilution and titration of cell-cycle regulators may control cell size in budding yeast
Source: PLoS Comput Biol. 2018 Oct 24;14(10):e1006548. doi: 10.1371/journal.pcbi.1006548 (PMC6218100; doi:10.1371/journal.pcbi.1006548)
Supplement: S2 Table — (DOCX) [file pcbi.1006548.s013.docx]

| **S2 Table. Parameters specific to the inhibitor-dilution model.** | | | |
| --- | --- | --- | --- |
| **Parameter** | **Description** | **Value** | **Unit**^a^ |
| $k_{\mathrm{WhiSbf}}^{\mathrm{As}}$ | association of Whi5 and SBF | 100 | AV/(AU$\cdot$min) |
| $k_{\mathrm{WhiSbf}}^{\mathrm{Ds}}$ | dissociation of Whi5:SBF complexes | 0.01 | 1/min |
| $k_{\mathrm{WhiClb}}^{\mathrm{Ph}}$ | Clb1/2-dependent Whi5 phosphorylation | 20 | AV/(AU$\cdot$min) |
| $k_{\mathrm{WhiCln}}^{\mathrm{Ph}}$ | Cln1/2-dependent Whi5 phosphorylation | 4 | AV/(AU$\cdot$min) |
| $k_{WhiCln3}^{\mathrm{Ph}}$ | Cln3-dependent Whi5 phosphorylation | 0.3 | AV/(AU$\cdot$min) |
| $k_{Cln3}^{\mathrm{Sy}}$ | Cln3 synthesis | 10 | AU/(molecule$\cdot$min) |
| $k_{\mathrm{Whi}}^{\mathrm{Sy}}$ | Whi5 synthesis | 0.016 | AU/(molecule$\cdot$min) |

^a^AU, arbitrary unit of number of molecules; AV, arbitrary unit of cell volume.
