# Supplementary material for: Dilution and titration of cell-cycle regulators may control cell size in budding yeast
Source: PLoS Comput Biol. 2018 Oct 24;14(10):e1006548. doi: 10.1371/journal.pcbi.1006548 (PMC6218100; doi:10.1371/journal.pcbi.1006548)
Supplement: S3 Table — (DOCX) [file pcbi.1006548.s014.docx]

| **S3 Table. Parameters specific to the titration-of-nuclear-sites model.** | | | |
| --- | --- | --- | --- |
| **Parameter** | **Description** | **Value** | **Unit**^a^ |
| $k_{\mathrm{WhiSbf}}^{\mathrm{As}}$ | association of Whi5 and SBF | 1 | AV/(AU$\cdot$min) |
| $k_{Cln3Whi}^{\mathrm{As}}$ | association of Whi5 and Cln3 | 100 | AV/(AU$\cdot$min) |
| $k_{Cln3Whi}^{\mathrm{Ds}}$ | dissociation of Cln3:Whi5 complexes | 0.1 | 1/min |
| $k_{\mathrm{WhiCln}}^{\mathrm{Ph}}$ | Cln1/2-dependent phosphorylation of free Whi5 | 100 | AV/(AU$\cdot$min) |
| $k_{WhiCln3}^{\mathrm{Ph}}$ | Whi5 phosphorylation in Cln3:Whi5:SBF complexes | 1 | 1/min |
| $k_{\mathrm{WhipCln}}^{\mathrm{Ph}}$ | Cln1/2-dependent hyper-phosphorylation of hypo-phosphorylated Whi5 in Whi5:SBF complexes | 3 | AV/(AU$\cdot$min) |
| $k_{WhipCln3}^{\mathrm{Ph}}$ | Cln3-dependent hyper-phosphorylation of hypo-phosphorylated Whi5 in Whi5:SBF complexes | 1 | AV/(AU$\cdot$min) |
| $k_{Cln3}^{\mathrm{Sy}}$ | Cln3 synthesis | 1.5 | AU/(molecule$\cdot$min) |
| $k_{\mathrm{Whi}}^{\mathrm{Sy}}$ | Whi5 synthesis | 0.02 | AU/(molecule$\cdot$min) |
| $NS_{t}$ | total number of nuclear sites (occupied by SBF) | 1^b^ | AU |

^a^AU, arbitrary unit of number of molecules; AV, arbitrary unit of cell volume.

^b^Parameter corresponds to a haploid cell. Changes made for ploidy mutants are listed in S4 Table.
