# Supplementary material for: Dilution and titration of cell-cycle regulators may control cell size in budding yeast
Source: PLoS Comput Biol. 2018 Oct 24;14(10):e1006548. doi: 10.1371/journal.pcbi.1006548 (PMC6218100; doi:10.1371/journal.pcbi.1006548)
Supplement: S4 Table — (DOCX) [file pcbi.1006548.s015.docx]

| **S4 Table. Non-zero initial conditions for both models**^a^**.** | | |
| --- | --- | --- |
| **Variable** | **Inhibitor-dilution model**^b^ | **Titration-of-nuclear-sites model**^b^ |
| $V_{m}$ | 1.6 AV | 1.2 AV |
| $GITM$ | 1 molecule | 1 molecule |
| $GDTM$ | 80 molecules | 60 molecules |
| $WHI$ | 1.6 AU | 4 AU |
| $WHI_{p}$ | 1.3 AU | 0.1 AU |
| $WHISBF$ | 1.6 AU | 0.66 AU |
| $CLN3$ | 1.6 AU | — |
| $CLN3WHISBF$ | — | 0.17 AU |
| $WHIpSBF$ | — | 0.17 AU |
| $CDH_{a}$ | 1.6 AU | 1.2 AU |
| $CDC_{i}$ | 1.6 AU | 1.2 AU |

^a^Initial conditions correspond to an average new-born haploid cell after budding.

^b^AU, arbitrary unit of number of molecules; AV, arbitrary unit of cell volume.
