# Supplementary material for: Dilution and titration of cell-cycle regulators may control cell size in budding yeast
Source: PLoS Comput Biol. 2018 Oct 24;14(10):e1006548. doi: 10.1371/journal.pcbi.1006548 (PMC6218100; doi:10.1371/journal.pcbi.1006548)
Supplement: S5 Table — (DOCX) [file pcbi.1006548.s016.docx]

| **S5 Table. Parameters changes for ploidy mutants.** | | | | | |
| --- | --- | --- | --- | --- | --- |
| **Mutant** | **Parameters**^a^ | | | | |
| 1$\times$*WHI5* haploid  1$\times$*CLN3* haploid | $GW_{t}=1$, | $GC_{t}=1$, | $GD_{t}=500$, | $GCN=1$, | $NS_{t}=1$ |
| 2$\times$*WHI5* haploid | $GW_{t}=2$, | $GC_{t}=1$, | $GD_{t}=500$, | $GCN=1$, | $NS_{t}=1$ |
| 1$\times$*CLN3* diploid | $GW_{t}=2$, | $GC_{t}=1$, | $GD_{t}=1000$, | $GCN=2$, | $NS_{t}=2$ |
| 1$\times$*WHI5* diploid | $GW_{t}=1$, | $GC_{t}=2$, | $GD_{t}=1000$, | $GCN=2$, | $NS_{t}=2$ |
| 2$\times$*WHI5* diploid  2$\times$*CLN3* diploid | $GW_{t}=2$, | $GC_{t}=2$, | $GD_{t}=1000$, | $GCN=2$, | $NS_{t}=2$ |

^a^All parameters are used in both models, except $NS_{t}$, which is only required for the titration model.
